# Marriage, Want, and Crime

**Published:** 1857-11

**Authors:** 


### MARRIAGE, WANT, AND CRIME

ARE not common associates. The celibate, the ignorant, and the idle, these are they who fill our prisons and crowd our penitentiaries. The more children a man has, the less apt is he to become a criminal. Each child is an additional responsibility. But each one touches some new chord in our affections, brings into exercise some new virtue, and calls out sentiments that otherwise might have remained dormant for a lifetime. One child is courageous, another timid; one is frank and open, another retires within itself; and thus each one has some quality or other, which, for that quality, makes it peculiarly loved; thus bringing out, one after another, every warm affection of a parent's heart, keeping his better nature always alive, steadily repelling temptation, and inviting to deeds of kindness and humanity. That all this is no mere theory, is evident from the fact, that four-fifths of the inmates of one of the largest penitentiaries in the Union were unable to read or write; of twenty-four hundred prisoners, only one out of six was married, and these averaged less than three children each. And then, it is an observed fact in great cities, that more persons are sent to prison during the abundance of summer than during winter, when want of fire and food combine to aggravate the sufferings of the poor.

These things being so, every good citizen should feel it his duty to encourage marriage, promote industry, and do all that is possible to educate the masses, not merely in the A B C, the reading, writing, and arithmetic, but in what is of higher moment than these, a familiar knowledge of those principles of human conduct which are so broadly and so plainly laid down in the Holy Scriptures of the Old and New Testament. Then, and not till then, will our physical and moral natures be found going hand in hand unto perfection.

